# Investigating the prevalence of problematic substance use and mental disorders in a large sample of prisoners with mental illness: network analysis

**DOI:** 10.1192/bjo.2023.514

**Published:** 2023-07-06

**Authors:** Nora van Buitenen, Jesse Meijers, Chantal J. W. van den Berg, Joke M. Harte

**Affiliations:** Judicial Complex Zaanstad, Dutch Custodial Institutions Agency, Ministry of Justice and Security, The Netherlands; and Department of Criminal Law and Criminology, Vrije Universiteit Amsterdam, The Netherlands; Judicial Complex Zaanstad, Dutch Custodial Institutions Agency, Ministry of Justice and Security, The Netherlands; and Willem Pompe Institute for Criminal Law and Criminology, Utrecht University, The Netherlands; Department of Criminal Law and Criminology, Vrije Universiteit Amsterdam, The Netherlands

**Keywords:** Forensic psychiatry, criminal offending, substance use, network analysis, prisoners with mental illness

## Abstract

**Background:**

The relationship between psychopathology and criminal offending has been the subject of many studies. Co-occurring substance use seems to increase the risk of offending in those with mental illness.

**Aims:**

To present data on the prevalence of mental disorders and demographics of prisoners with mental illness, and investigate associations between diagnoses and substance use from a network perspective.

**Method:**

Data used in this study are part of a cohort study within the four penitentiary psychiatric centres in The Netherlands. It includes data of 4956 incarcerated male patients. Prevalence rates of mental disorders and demographic variables were compared between individuals with and without problematic substance use. A network of diagnoses, including three categories of substance use, was constructed with regression coefficients.

**Results:**

Most patients showed prior problematic substance use (72.2%) in more than one category of substances (58.7%). Problematic substance use was associated with diagnoses of schizophrenia spectrum disorders (*χ^2^*(1) = 37.52, *P* < 0.001, *V* = 0.09) and cluster B personality disorders (*χ^2^*(1) = 56.39, *P* < 0.001, *V* = 0.11). Three major findings of the network are discussed in detail: the role of antisocial personality disorder, impulsivity and psychotic disorders in combination with problematic substance use.

**Conclusions:**

Problematic substance use is highly prevalent among prisoners with mental illness, and should always be taken into account in research on this topic. Treatment should target substance use to reduce the risk of recidivism. Further differentiation in categories of substances is needed for the development of risk profiles.

During the past several decades, the relationship between substance use and risk of (violent) criminal offending has been the subject of many studies. The risk of offending increases for individuals misusing substances,^1^ making it a widely accepted risk factor for criminal behavior.^2^ However, research indicates that the relationship between substance use and criminal offending is not the same for every type of drug.^3^ Drugs commonly associated with offending are heroin, crack and cocaine.^1,4^ Furthermore, there is ample evidence for an association between alcohol use and (violent) criminal offending.^5,6^ A complicating factor in the relationship between the use of specific types of substances and criminal behaviour, is the fact that individuals struggling with addiction often use several types of substances.^7,8^ Research has shown that polysubstance use increases the risk of criminal offending above and beyond the effects of single substance use,^9,10^ indicating that the phenomenon is more than the sum of its parts.

## Comorbidity

Individuals who were diagnosed with a serious mental illness seem to be more prone to co-occurring problematic substance use than those who were not.^7,11,12^ In a review of 22 meta-analyses investigating the risk of violence associated with psychiatric diagnoses, comorbid substance use disorder (SUD) appeared to be the strongest risk factor for violent behaviour in people with a psychiatric disorder.^13^ Comorbid substance use increases the risk of offending in patients with schizophrenia,^14^ bipolar disorder,^15^ attention-deficit hyperactivity disorder (ADHD)^16^ and, possibly, autism spectrum disorder.^17,18^ It has been suggested that the increased risk of violence and criminal offending by individuals with mental illness are, at least in part, a result of comorbid SUDs.^5,19^

Although there are meaningful associations between problematic substance use, serious mental illness and criminal offending, the direction of these associations remains unclear, and it seems plausible that they are not unilateral. Furthermore, the associations might differ across drug types and diagnoses.^5,20^ Differentiating between drug types could provide hypotheses on specific risk profiles for criminal offending by individuals with mental illness, as certain combinations of psychopathology and substance use might put individuals at an elevated risk for offending behaviour. Additionally, associations could be obscured by polysubstance use and psychiatric comorbidity in offenders with mental illness.^21^ Most research regarding this topic has focused on the mediating effect of co-occurring substance use on criminal offending for a single disorder or single class of disorders,^22^ without accounting for the effects of other comorbid disorders and polysubstance use.

## Study aims

The current study aims to provide a detailed, overarching view of the relationships between mental illnesses and problematic substance use in a population of prisoners. First, data on the prevalence of mental disorders and other demographic variables in a sample of prisoners with mental illness (*N* = 4956) with and without problematic substance use are presented and compared. Second, by using a network approach,^23^ associations between DSM diagnoses and problematic substance use are investigated (*n* = 3578). This technique will allow us to visually explore the associations between mental disorders and problematic substance, as well as differentiate between categories of substance use. Three major categories are included in the model: alcohol use, the use of cannabis and the use of hard drugs. By using these categories, the model will account for patients’ use of substances in multiple categories. The results of this study will contribute to our understanding of the associations between problematic substance use and mental disorders in offender populations.

## Method

### Penitentiary psychiatric centres and the National PPC Database

The present study uses data collected in the penitentiary psychiatric centres (PPCs) in The Netherlands. PPCs are facilities within the Dutch penitentiary system, housing detainees incapable of functioning within a regular prison regime because of their mental state. Since 1 May 2013, PPCs are required to systematically gather information on patients admitted, resulting in the National PPC Database. The database contains diagnostic information, demographics and patient characteristics, and criminal records. The data are primarily used for policy making. For scientific research, the data are available in an anonymised version.

### Ethical considerations

The authors assert that all procedures contributing to this work comply with the ethical standards of the relevant national and institutional committees on human experimentation and with the Helsinki Declaration of 1975, as revised in 2008. Secondary use of the previously gathered, anonymised data was authorised by the Dutch Ministry of Justice and Security. Additionally, the research plan was presented to the Ethics Committee of the Department of Law and Criminology (in Dutch: Facultaire Commissie Ethiek Rechtswetenschappelijk & Criminologisch Onderzoek: CERCO), Vrije Universiteit Amsterdam. The committee had no ethical objections and positively advised on the research plan on 16 January 2020.

### Participants

This study includes data on patients detained in a PPC between 1 May 2013 and 3 September 2020. In cases of multiple admissions, data gathered during the most recent admission were included. A sample of 6802 individuals was identified. Given possible gender differences in comorbidity^24^ and problematic substance use,^25^ female patients were excluded (*n* = 610). Finally, because of insufficient reliable sources of information, the history of substance use (problematic or not) could not be determined for 1236 patients, resulting in a sample of 4956 patients. The criminal status of 3469 patients (70%) was defined as pre-trial detention upon admission to the PPC; all other patients were convicted.

### Measures

#### DSM diagnosis

Upon admission to the PPC, both a psychiatrist and a psychologist conduct an independent primary interview with the patient. The final DSM diagnosis is the result of a consensus diagnosis between these two professionals. For the network analysis, the numerous DSM diagnoses had to be categorised into broader categories. See Supplementary Appendix 1 available at https://doi.org/10.1192/bjo.2023.514 for a detailed account of this categorisation. Categories with an insufficient sample size (*n* < 20), which could not be merged without compromising their clinical relevance, were excluded from the network analysis.

A new edition of the DSM was published within the duration of this study, therefore diagnoses were based on either the DSM-IV or DSM-5. Both were recoded into the corresponding ICD-9-CM code. For a detailed description on the merging of DSM-IV and DSM-5 diagnoses, see Supplementary Appendix 1.

#### Demographics

Several demographic measures were examined, including age at admission, criminal history, level of education and country of birth following the definition of ethnic groups as presented by the Dutch Central Bureau of Statistics.^26^

#### Problematic substance use

The presence of problematic substance use was not assessed by means of the DSM diagnoses of SUD. A substantial part of the sample could have been incarcerated before admission to the PPC, forcing a possible SUD into remission, which would then be missed upon admission. Furthermore, SUDs are at risk of being underdiagnosed in cases of florid psychosis, a mental state that applies to many patients upon admission to the PPC.^21^

Therefore, the presence of problematic substance use was assessed with the Dutch Historisch, Klinisch, Toekomst – Revisie scale (which translates as ‘Historical, Clinical, Future – Revised’; HKT- R^27^) The HKT-R consists of 33 risk factors for (violent) offending and is systematically scored for all patients admitted to the PPC. Item H10, ‘History of addiction’, was used to assess the lifetime presence of problematic substance use. Scoring was based on all criminal files available to the researchers, often including extensive psychological reports. If insufficient reliable sources of information were available to assess a lifetime presence of problematic substance use, the item was scored as missing; for example, when individuals spent prolonged periods of time abroad or had recently migrated to The Netherlands. The item was scored on a five-point Likert scale (0–4). A score of either 3 (frequent problems such as various instances of financial or housing issues, verbal aggression or disorderly conduct as a result of substance use) or 4 (expressions of physical violence as a result of substance use) was defined as problematic substance use. The item is scored separately for the use of alcohol, cannabis and hard drugs. For a description of specific substances that comprise the hard drugs category, see Supplementary Appendix 2. The historical subscale, to which H10 belongs, has good internal consistency, with a Cronbach's alpha of 0.80. Furthermore, item H10 has shown to have a high intraclass correlation of 0.83, which is a measure of the interrater reliability.^27^

### Statistical analyses

Descriptive statistics and prevalence rates were calculated with SPSS version 27 for Windows (IBM, New York, USA). Comparisons of comorbid mental disorders between individuals with and without problematic substance use were performed by using either chi-squared tests or *t*-tests. For significantly associated variables, effect sizes were calculated with Cramer's *V*-statistic and Cohen's *d*-statistic, respectively. Furthermore, a Bonferroni correction was applied in all analyses.

Network analyses were conducted in R version 4.1.2 for macOS (R Foundation for Statistical Computing, Vienna, Austria; see https://www.R-project.org/). Because of the binary nature of the data, the ‘IsingFit’ package (version 0.3.1)^28,29^ was used to estimate the network parameters. Based on logistic regressions, the best-fitting function was selected by using the extended Bayesian information criterion, which has proven to estimate the most relevant features of a network successfully. To ensure sparsity of the network and cope with the problem of multicollinearity and multiple testing, all regression coefficients were penalised with the Enhanced Least Absolute Shrinkage and Selection Operator Regression Model (eLasso), resulting in more conservative network structures. The hyperparameter was set to 0.25.^29,30^ The resulting matrix was plotted with the ‘qGraph’ package (version 1.9.2).^31^ The edges represent the associations between variables and can be plotted using either an ‘AND rule’ (i.e. reciprocal relationships) or an ‘OR rule’ (i.e. one-sided relations). The current paper will provide a figure of the model using the ‘OR rule’. Only positive estimates were included in the model to clearly describe associations between diagnoses and substance use, as comorbidity is indicated by positive edges. In the visualisation of the network, the Fruchterman–Reingold algorithm was used, which places strongly connected nodes close to each other.^32^

## Results

### Prevalence of mental disorders

In total, 72.2% of the sample had a history of problematic substance use (*n* = 3578), of whom 65.4% problematically used alcohol (*n* = 2274), 62.0% problematically used cannabis (*n* = 2130) and 59.8% problematically used hard drugs (*n* = 2058). Most patients (58.7%) used substances in more than one category. In total, 36.9% used two categories of substances (*n* = 1320), and 21.9% used all three categories of substances (*n* = 782). Prevalence rates of mental disorders are displayed in Table 1. Individuals with problematic substance use were significantly more often diagnosed with schizophrenia spectrum and other psychotic disorders (*χ^2^*(1) = 37.52, *P* < 0.001, *V* = 0.09), and cluster B personality disorders (*χ^2^*(1) = 56.39, *P* < 0.001, *V* = 0.11), although effect sizes were small. All other significant results in Table 1 indicate that individuals without substance use problems were more often diagnosed with the disorders listed in the table than those with substance use problems.

**Table 1:** Prevalence of mental disorders in individuals with and without problematic substance use (*N* = 4956)

	No problematic substance use (*n* = 1378)		Problematic substance use (*n* = 3578)	
	*n*	%	*n*	%
Deferred diagnosis axis 1**	50	3.6	57	1.6
Deferred diagnosis axis 2**	406	29.5	881	24.6
Neurodevelopmental disorders	279	20.2	736	20.6
Schizophrenia spectrum and other psychotic disorders**	672	48.8	2090	58.4
Bipolar and related disorders	57	4.1	167	4.7
Depressive disorders**	115	8.3	136	3.8
Anxiety disorders**	24	1.7	31	0.9
Obsessive–compulsive and related disorders*	14	1.0	15	0.4
Trauma- and stressor-related disorders**	150	10.9	237	6.6
Dissociative disorders**	5	0.4	1	0.0
Somatic symptom and related disorders**	11	0.8	7	0.2
Feeding and eating disorders	0	0.0	3	0.1
Elimination disorders	1	0.1	0	0.0
Sleep–wake disorders	2	0.1	3	0.1
Sexual dysfunctions	1	0.1	0	0.0
Gender dysphoria	1	0.1	5	0.1
Disruptive, impulse-control and conduct disorders*	51	3.7	85	2.4
Neurocognitive disorders	35	2.5	84	2.3
Paraphilic disorders**	55	4.0	19	0.5
Other mental disorders**	33	2.4	40	1.1
Movement disorders and other side-effects	0	0.0	2	0.1
Cluster A (odd or eccentric disorders)	9	0.7	23	0.6
Cluster B (dramatic, emotional or erratic disorders)**	101	7.3	550	15.4
Cluster C (anxious or fearful disorders)*	15	1.1	18	0.5
Other/unspecified personality disorder	183	13.3	540	15.1

* *P* < 0.05, **P < 0.001.

### Demographics

Information on demographic variables is shown in Table 2. On average, individuals with problematic substance use (mean 35.94, s.d. = 10.12) were significantly younger at admission (*t*(2043.51) = 2.77, *P* = 0.002, *d* = 0.11) than those without problematic substance use (mean 37.02, s.d. = 13.08). The proportion of individuals completing a primary education was smaller for those without problematic substance use. Alternatively, the proportion of individuals completing a vocational or higher-level education was smaller for those with problematic substance use (*χ²*(5) = 176.48, *P* < 0.001, *V* = 0.19). Individuals with and without problematic substance use differed significantly with regard to country of birth (*χ²*(11) = 20.79, *P* < 0.001, *V* = 0.06). The proportion of individuals born in Morocco was larger for those with problematic substance use. Furthermore, results show that individuals without problematic substance use were more likely to be first offenders and those with problematic substance use were more likely to be repeat offenders (*χ²*(1) = 367.02, *P* < 0.001, *V* = 0.27). Finally, individuals with and without problematic substance use differed in the categories of offence they were detained for (*χ²*(13) = 272.71, *P* < 0.001, *V* = 0.24). Most notable is that the proportion of individuals detained for (violent) property offences was larger for those with problematic substance use. It should be noted that effect sizes are small to medium.

**Table 2:** Demographics of patients with and without problematic substance use (*N* = 4956)

	No problematic substance use (*n* = 1378)	Problematic substance use (*n* = 3578)
Mean age, years* (s.d.)	37.02 (13.08)	35.94 (10.12)
Education, *n* (%)		
None	62 (4.5)	168 (5.2)
Primary education*	323 (23.5)	136 (38.3)
Secondary education	418 (30.4)	1019 (28.6)
Vocational education*	226 (16.4)	423 (11.9)
University of applied science and higher*	109 (7.9)	77 (2.2)
Country of birth, *n* (%)		
The Netherlands	893 (64.8)	2304 (64.4)
Other non-Western	194 (14.1)	462 (12.9)
Other Western	112 (8.1)	266 (7.4)
Morocco*	39 (2.8)	169 (4.7)
Suriname	50 (3.6)	123 (3.4)
The Netherlands Antilles	57 (4.1)	165 (4.6)
Turkey	29 (2.1)	80 (2.2)
Recidivism, *n* (%)		
First offence*	302 (22.0)	156 (4.4)
Reoffence*	1071 (78.0)	3418 (95.6)
Offence category, *n* (%)		
Minor offence	26 (1.9)	71 (2.0)
Drug offence*	33 (2.4)	55 (1.5)
Destruction of property*	21 (1.5)	87 (2.4)
Property offence*	131 (9.5)	691 (19.3)
Moderate violent offence	384 (27.9)	999 (27.9)
Severe violent offence	116 (8.4)	303 (8.5)
Violent property offence*	92 (6.7)	411 (11.5)
Sexual offence*	75 (5.4)	124 (3.5)
Paedosexual offence*	87 (6.3)	29 (0.8)
Manslaughter*	187 (13.6)	373 (10.4)
Arson	60 (4.4)	193 (5.4)
Murder*	158 (11.5)	226 (6.3)

* *P* < 0.05.

### Network of problematic substance use and comorbid mental disorders

**Fig. 1:**
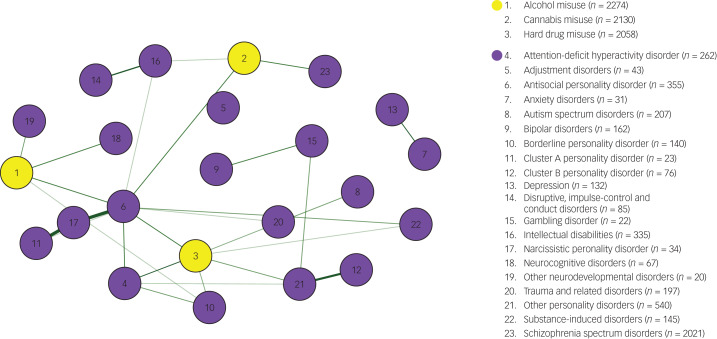
Network model of DSM diagnoses and problematic substance use (*N* = 3578). *Note:* Yellow nodes indicate categories of problematic substance use; purple nodes indicate categories of mental disorders. The thickness of the green edges represents the strength of the positive correlation, with thicker lines representing stronger correlations.

The final network model (Fig. 1) includes data on DSM diagnoses and the use of substances of all 3578 patients with a history of problematic substance use. Thicker edges represent stronger correlations. Node names and sample sizes are listed in the legend.

The node that represents the problematic use of hard drugs has the most associations of the included categories of substances. It is associated with antisocial personality disorder (ASPD), borderline personality disorder (BPD), ADHD, other personality disorders, autism spectrum disorder and substance-induced disorders. These last two associations disappear when the more conservative ‘AND rule’ is applied. This indicates that the associations are unidirectional.

The problematic use of cannabis is also associated with ASPD. Furthermore, it is associated with the node that represents schizophrenia spectrum disorders. Finally, the use of cannabis is associated with intellectual disabilities, but only when the ‘OR rule’ is applied.

Problematic use of alcohol is associated with ASPD. Furthermore, the problematic use of alcohol is associated with neurocognitive disorders, other neurodevelopmental disorders and BPD, but only when the ‘OR rule’ is applied.

The three categories of substances do not share a direct association; they are indirectly associated through the node that represents ASPD.

## Discussion

This study presents data on the prevalence of mental disorders and demographic variables in a large sample of prisoners with mental illness, comparing individuals with and without problematic substance use. The results show that a considerable number, over 70%, had a history of problematic substance use, with a majority misusing substances in multiple categories. Those with problematic use were more often detained for (violent) property offences and showed higher proportions of recidivism. These findings underline the very relevant role of substance misuse in the development of patterns of offending behaviour by individuals with mental illness. Crimes that are known to have a high rate of recidivism, such as burglary and robbery,^33^ are more prevalent among individuals with problematic substance use, and offenders of such crimes are even more likely to reoffend if they have a history of drug misuse.^34^

Individuals with mental illness who exhibit criminal behaviour are likely to be committed to closed facilities, forcing them into abstinence. In doing so, the interactions between the psychiatric disorder and the comorbid SUD(s) are reduced during treatment in these facilities. However, without appropriate treatment also targeting the problematic use of substances, they are likely to relapse upon release. This relapse could result in a drastic increase in psychiatric symptoms and an (immediate) increased risk of criminal behavior.^2,5^ Taken together, treating substance use problems in these dually diagnosed offenders is probably an effective strategy when it comes to reducing recidivism rates. Few treatment programmes have been developed specifically to target substance use in offenders with mental illness, and although these programmes seem promising, they need further evaluation.^35,36^

The main focus of this study was to provide an overarching view of the associations between psychiatric diagnoses and three major categories of problematic substance use in a large sample of offenders with mental illness. Analyses revealed many interesting connections, but three findings seem to stand out: the role of ASPD, the role of impulsivity and the role of psychotic disorders in combination with problematic use of cannabis.

### The role of ASPD

One finding seems to be of particular interest when investigating combinations of mental disorders with problematic substance use that could increase the risk of offending behaviour: ASPD is connected to all three categories of substances, and plays a central role in the network presented in the current study.

Research has shown that ASPD is a potent predictor of criminal offending.^37^ Moreover, criminal offending is included as a diagnostic criterion of the disorder.^38^ It seems plausible that the risk of offending by individuals with ASPD is mediated by problematic substance use; for example, by increasing already high levels of impulsivity and aggression.^20,39^ Also, the risk of offending behaviour in individuals with ASPD increases when intoxicated.^40^ Another possibility is that ASPD is a confounding variable, increasing the risk of offending in both severe mental illness and problematic substance use.^41^ Although the current study cannot clarify the direction or underlying mechanism of the association between ASPD and SUDs, it does indicate that there is an effect that generalises across the three substance categories.

Another interesting finding in the current study is that none of the substance categories were directly connected within the network model, but connected to each other through ASPD even when using the most conservative methods of analysis. This indicates that ASPD plays a central role in explaining the high prevalence of polysubstance use in offenders with mental illness.

### The role of impulsivity

The problematic use of hard drugs is another key feature of the network presented in this study, as it is associated with the highest number of mental disorders compared with alcohol and soft drug use. Given the associations with ASPD, BPD and ADHD, we argue that impulsivity is the underlying construct responsible for these associations.^21^ Impulsivity is shown to significantly predict problematic substance use,^42^ aggression^43^ and delinquency.^44^ The results of the current study identify a specific association of impulsivity with problematic use of hard drugs as a potential combination that puts individuals at risk for criminal offending.

Although the main body of empirical studies yields mixed results,^8^ it has been proposed that individuals with ADHD are prone to self-medicating their symptoms by using stimulants, because of the calming effect that stimulants have on them.^45^ If impulsivity is, in fact, the driving force in these associations, one could hypothesise that impulsive individuals perhaps have similar preferences in the substance they use. More precise differentiation, using more specific categories of substance use, is needed to further explore this association and its relation to criminal offending.

### Psychotic disorders and problematic use of cannabis

There is little doubt about the existence of an association between psychotic disorders and substance use,^7,46^ and substance use has a mediating effect on the risk of (violent) crime by individuals with a schizophrenia spectrum disorder.^5,47^ In the current study, the problematic use of cannabis is the only variable associated with psychotic disorders; this seems to be in line with research indicating that cannabis is the drug that is most often used by individuals with psychotic disorders.^46^ It seems likely that this connection between cannabis use and psychotic disorders puts individuals at risk for criminal behaviour. A pathway for this association has been proposed: the use of cannabis might induce or magnify positive symptoms, which increases the risk for offending.^48^

### Strengths and limitations

As mentioned, most studies investigating the associations between mental disorders, comorbid substance use and criminal offending focus on the effects within a single disorder, and research on this topic is often complicated by polysubstance use. This study presents data on a large sample of prisoners with mental illness, includes a wide range of mental disorders and three major categories of problematic substance use. Modelling these variables within a single model with a network approach offers two main advantages. First, possible obscuring effects of polysubstance use are discounted. Second, possible effects of other comorbid mental disorders are accounted for. The current study provides a representation of the complex psychopathology within this population, which is sensitive to the effects of high rates of polysubstance use and comorbidity.

This study entails a secondary analysis of a large body of data gathered in clinical practice. Although diagnoses were carefully made, no standardised diagnostic process for research purposes was used to establish the diagnoses. Furthermore, although the diagnoses were established as present upon admission, problematic substance use was established as present during the patients’ lifetime. This discrepancy in temporality of these measures could be viewed as a limitation. The consideration for using the HKT-R item H10 instead of DSM diagnoses is explained in the Method section. Using DSM diagnoses of SUDs upon admission would result in more significant methodological limitations. Problematic substance use is often a persistent, long-lasting problem in this population, and use of the lifetime presence of problematic substance use provides, in our view, a more accurate representation of problematic substance use. It should be noted that item H10 does not define whether a crime was committed under the influence of a substance. The current study does not aim to make claims about the state of mind a crime was committed in.

Finally, the data did not allow for further specification of the substances, which would have contributed substantially, especially given the significant effect of problematic hard drug use.

### Future research

The results yielded by this study again underline the high prevalence of problematic substance use in offenders with mental illness. This combination of psychopathology poses a challenge for forensic healthcare professionals. Therefore, the development of treatment programmes for offenders with mental illness with co-occurring problematic substance use is of much importance. Adequate treatment for these offenders could reduce the risk of recidivism.

Furthermore, this study investigated combinations of mental disorders and problematic use of substances that could increase the risk of criminal offending. Several directions for future research follow from this. The combination of ASPD and problematic substance use and its effects on criminal offending should be further investigated, not only focusing on assessing the cumulative risk of combining these factors, but also aiming to shed light on underlying mechanisms. Such research may become possible if and when the alternative diagnostic model for personality disorders, as proposed in part III of the DSM-5, becomes the new standard.^38^ More specific information on pathological traits, such as impulsivity, will then become available in data-sets.

Finally, other lines of research should revolve around the need for further differentiation based on more specific categories of problematic substance use, to develop more specific risk profiles. Further specifications of the role of impulsivity in the use of hard drugs, and the association between cannabis use and psychotic disorders, could contribute to adequately assessing the risk of future criminal offending by offenders with mental illness, and could aid in the detection of individuals at risk for future offending.

## Data Availability

The authors received permission from the Dutch Ministry of Justice and Security to access the data used in this study. However, they are unable to share the data as they are not the data custodians. Permission to conduct scientific research within a specific division of the Dutch Custodial Institutions Agency must be requested from the director of the relevant sector. Further information can be found online at https://www.dji.nl/over-dji/leren--onderzoek/wetenschappelijk-onderzoek-bij-dji.

## Supplementary material

For supplementary material accompanying this paper visit http://doi.org/10.1192/bjo.2023.514. click here to view supplementary material
