# Supplementary material for: Investigating the prevalence of problematic substance use and mental disorders in a large sample of prisoners with mental illness: network analysis
Source: BJPsych Open. 2023 Jul 6;9(4):e122. doi: 10.1192/bjo.2023.514 (PMC10375871; doi:10.1192/bjo.2023.514)
Supplement: Supplementary file 1 [file bjosup.zip › S2056472423005148sup001.docx]

**Appendix A**

As of January 1, 2017 the DSM-5 is used to assess the presence of a mental disorder in the Netherlands. Before this date the DSM-IV was used for this purpose. Due to this change in classifications systems, the data used in this study consists of both DSM-IV and DSM-5 scores. For all subjects included in this study, ICD-9 codes were available. The ICD-9 codes were linked to specific mental disorders and their decription in either DSM-IV, DSM-5 or both if the code appeared in both versions.

The linked codes were examined by trained professionals, both in the field of forensic psychiatry and forensic psychology, and recoded in overarching categories ensuring both clinical relevance of the categories and a sufficient sample size within them for further analysis. Most ICD-9 codes were present in both DSM-IV and DSM-5 and had a similar dispriction in both DSM versions. If the ICD-9 code referenced to fundamentally different discriptions of mental disorders within the versions of the DSM, we indicated to which version of the DSM the ICD-9 code included in the category refers. Resulting in the following 23 categories:

**Attention-Deficit/Hyperactivity Disorder**

314.00 ADHD, predominantly inattentive presentation

314.01 ADHD, combined presentation

ADHD, predominantly hyperactive/impulsive presentation

Other specified ADHD

Unspecified ADHD

314.90 Attention-deficit/hyperactivity disorder NOS

**Autism Spectrum Disorders**

299.00 Autistic disorder

Autism spectrum disorder

299.80 Rett's disorder

Asperger’s disorder

Pervasive developmental disorder NOS

**Other Neurodevelopmental Disders**

307.00 Adult-onset fluency disorder

Stuttering

307.20 Unspecified tic disorder

Other specified tic disorder

Tic disorder NOS

307.22 Persistent (chronic) motor or vocal tic disorder

307.23 Tourette’s disorder

307.90 Unspecified communication disorder

Communication disorder NOS

315.00 Specific learning disorder, with impairment in reading

Learning disorder

315.10 Specific learning disorder, with impairment in mathematics

Mathematics disorder

315.39 Language disorder

Social (pragmatic) communication disorder

Speech sound disorder

Phonological disorder

315.80 Other specified neurodevelopmental disorder

Global developmental delay

315.90 Unspecified neurodevelopmental disorder

Stuttering

Learning disorder not otherwise specified

**Intellectual Disability**

317.00 Intellectual disability (intellectual developmental disorder), mild

Mild mental retardation

318.00 Intellectual disability (intellectual developmental disorder), moderate

Moderate mental retardation

318.10 Intellectual disability (intellectual developmental disorder), severe

Severe mental retardation

318.2 Intellectual disability (intellectual developmental disorder), profound

Profound mental retardation

319.00 Intellectual disability (intellectual developmental disorder)

Mental retardation; severity unspecified

**Schizophrenia Spectrum and Other Psychotic Disorders**

295.10 Schizophrenia disorganized type

295.20 Schizophrenia catatonic type

295.30 Schizophrenia paranoid type

295.40 Schizophreniform disorder

295.60 Schizophrenia residual type

295.70 Schizoaffective disorder, bipolar type

Schizoaffective disorder, depressive type

295.90 Schizophrenia

Schizophrenia undifferentiated type

297.10 Delusional disorder

298.80 Brief psychotic disorder

Other specified schizophrenia spectrum and other psychotic disorder

298.90 Unspecified schizophrenia spectrum and other psychotic disorder

Psychotic disorder NOS

**Bipolar Disorder**

296.00 Bipolar I disorder, single manic episode unspecified

296.01 Bipolar I disorder, single manic episode mild

296.02 Bipolar I disorder, single manic episode, moderate

296.04 Bipolar I disorder, single manic episode, sever with psychotic features

296.06 Bipolar I disorder, single manic episode, in full remission

296.40 Bipolar I disorder, current or most recent episode hypomanic

Bipolar I disorder, current or most recent episode hypomanic, unspecified

Bipolar I disorder, current or most recent episode manic, unspecified

296.41 Bipolar I disorder, current or most recent episode manic, mild

296.42 Bipolar I disorder, current or most recent episode manic, moderate

296.43 Bipolar I disorder, current or most recent episode manic, severe

Bipolar I disorder, current or most recent episode manic, severe without psychotic features

296.44 Bipolar I disorder, current or most recent episode manic, with psychotic features

296.45 Bipolar I disorder, current or most recent episode hypomanic, in partial

remission

Bipolar I disorder, current or most recent episode manic, in partial remission

296.46 Bipolar I disorder, current or most recent episode hypomanic, in full remission

Bipolar I disorder, current or most recent episode manic, in full remission

296.50 Bipolar I disorder, current or most recent episode depressed, unspecified

296.51 Bipolar I disorder, current or most recent episode depressed, mild

296.52 Bipolar I disorder, current or most recent episode depressed, moderate

296.53 Bipolar I disorder, current or most recent episode depressed, severe without

psychotic features

296.54 Bipolar I disorder, current or most recent episode depressed, with psychotic

features

296.60 Bipolar I disorder, most recent episode mixed unspecified

296.64 Bipolar I disorder, most recent episode mixed, severe with psychotic features

296.70 Bipolar I disorder, current or most recent episode unspecified

296.80 Unspecified bipolar and related disorder

Bipolar disorder NOS

296.89 Bipolar II disorder

Other specified bipolar and related disorder

296.90 Only if diagnosed under DSM-IV: Mood disorder NOS

**Depressive Disorders**

296.20 Major depressive disorder, single episode, unspecified

296.21 Major depressive disorder, single episode, mild

296.22 Major depressive disorder, single episode, moderate

296.23 Major depressive disorder, single episode, severe

Major depressive disorder, single episode, severe, without psychotic features

296.24 Major depressive disorder, single episode, with psychotic features

296.25 Major depressive disorder, single episode, in partial remission

296.26 Major depressive disorder, single episode, in full remission

296.30 Major depressive disorder, recurrent episode, unspecified

296.31 Major depressive disorder, recurrent episode, mild

296.32 Major depressive disorder, recurrent episode, moderate

296.33 Major depressive disorder, recurrent episode, severe

296.34 Major depressive disorder, recurrent episode, with psychotic features

296.35 Major depressive disorder, recurrent episode, in partial remission

296.36 Major depressive disorder, recurrent episode, in full remission

300.40 Persistent depressive disorder (dysthymia)

Dysthymic disorder

311.00 Unspecified depressive disorder

Other specified depressive disorder

Depressive disorder NOS

**Anxiety Disorders**

300.00 Unspecified anxiety disorder

Anxiety disorder

300.01 Panic disorder

Panic disorder withoout agoraphobia

300.02 Generalized anxiety disorder

300.21 Panic disorder with agoraphobia

300.22 Agoraphobia

Agoraphobia without history of panic disorder

300.23 Social anxiety disorder (social phobia)

300.29 Specific phobia, animal

Specific phobia, blood-injection-injury

Specific phobia, natural environment

Specific phobia, other

Specific phobia, situational

Specific phobia

**Trauma and Stressor related Disorders**

308.30 Acute stress disorder

309.81 Posttraumatic stress disorder

309.89 Other specified trauma- and stressor-related disorder

309.90 Unspecified trauma- and stressor-related disorder

Adjustment disorder, unspecified

313.89 Disinhibited social engagement disorder

Reactive attachment disorder

**Adjustment Disorder**

309.00 Adjustment disorder, with depressed mood

309.00 Adjustment disorder, with mixed disturbance of emotions and conduct

309.24 Adjustment disorder, with anxiety

309.28 Adjustment disorder, with mixed anxiety and depressed mood

309.30 Adjustment disorder, with disturbance of conduct

309.40 Adjustment disorder with mixed disturbance of emotions and conduct

**Neurocognitive Disorders**

290.40 Vascular neurocognitive disorder, probable, with/without behavioral disturbance

Vascular dementia uncomplicated with/without behavioral disturbance

294.10 Major frontotemporal neurocognitive disorder, probable, without behavioral

disturbance

Major neurocognitive disorder due to Alzheimer's disease, probable, without

behavioral disturbance

Major neurocognitive disorder due to another medical condition, without behavioral disturbance

Major neurocognitive disorder due to HIV infection, without behavioral

disturbance

Major neurocognitive disorder due to Huntington's disease, without behavioral disturbance

Major neurocognitive disorder due to multiple etiologies, without behavioral disturbance

Major neurocognitive disorder due to Parkinson's disease, Probable, without behavioral disturbance

Major neurocognitive disorder due to prion disease, without behavioral

disturbance

Diffuse traumatic brain injury with loss of consciousness of unspecified duration

Major neurocognitive disorder due to traumatic brain injury, without behavioral

disturbance

Major neurocognitive disorder with Lewy bodies, probable, without behavioral disturbance

Dementia of the Alzheimer’s Type, without behavioral disturbance

Dementia due to HIV disease, without behavioral disturbance

Dementia due to head trauma, without behavioral disturbance

Dementia due to Parkinson's disease, without behavioral disturbance

Dementia due to Huntington's disease, without behavioral disturbance

Dementia due to Pick’s disease, without behavioral disturbance

Dementia due to Creutzfeldt–Jakob disease, without behavioral disturbance

Dementia due to other general medical condition, without behavioral disturbance

294.11 Major frontotemporal neurocognitive disorder, probable, with behavioral

disturbance

Major neurocognitive disorder due to Alzheimer's disease, probable, with behavioral disturbance

Major neurocognitive disorder due to another medical condition, with behavioral

disturbance

Major neurocognitive disorder due to HIV infection, with behavioral disturbance

Major neurocognitive disorder due to Huntington's disease, with behavioral

disturbance

Major neurocognitive disorder due to multiple etiologies, with behavioral disturbance

Major neurocognitive disorder due to Parkinson's disease, probable, with behavioral disturbance

Major neurocognitive disorder due to prior disease, with behavioral disturbance

Major neurocognitive disorder due to traumatic brain injury, with behavioral disturbance

Major neurocognitive disorder with Lewy bodies, probable, with behavioral

disturbance

Dementia of the Alzheimer’s Type, with behavioral disturbance (early/late)

Dementia due to HIV disease, with behavioral disturbance

Dementia due to head trauma, with behavioral disturbance

Dementia due to Parkinson's disease, with behavioral disturbance

Dementia due to Huntington's disease, with behavioral disturbance

Dementia due to Pick’s disease, with behavioral disturbance

Dementia due to Creutzfeldt–Jakob disease, with behavioral disturbance

Dementia due to other general medical condition, with behavioral disturbance

294.90 Only if diagnosed under DSM-IV: Cognitive disorder not otherwise specified

331.83 Mild frontotemporal neurocognitive disorder

Mild neurocognitive disorder due to Alzheimer's disease

Mild neurocognitive disorder due to another medical condition

Mild neurocognitive disorder due to HIV infection

Mild neurocognitive disorder due to Huntington's disease

Mild neurocognitive disorder due to multiple etiologies

Mild neurocognitive disorder due to Parkinson's disease

Mild neurocognitive disorder due to prion disease

Mild neurocognitive disorder due to traumatic brain injury

Mild neurocognitive disorder with Lewy bodies

Mild vascular neurocognitive disorder

**Disruptive Behavior and Impulse-Control Disorders**

312.30 Impulse-control disorder NOS

312.33 Pyromania

312.34 Intermittent explosive disorder

312.81 Conduct disorder, childhood-onset type

312.82 Conduct disorder, adolescent-onset type

312.89 Conduct disorder, unspecified onset

Other specified disruptive, impulse-control, and conduct disorder

312.90 Unspecified disruptive, impulse-control, and conduct disorder

Disruptive behavior disorder NOS

313.81 Oppositional defiant disorder

**Substance Induced Disorders**

291.30 Alcohol induced psychotic disorder, with hallucinations

291.90 Alcohol induced psychotic disorder

292.11 Amphetamine induced psychotic disorder, with delusions

Cannabis induced psychotic disorder, with delusions

Cocaine induced psychotic disorder, with delusions

Hallucinogen induced psychotic disorder, with delusions

Inhalant induced psychotic disorder, with delusions

Opioid induced psychotic disorder, with delusions

Phencyclidine induced psychotic disorder, with delusions

Sedative, hypnotic, or anxiolytic induced psychotic disorder, with delusions

Other substance induced psychotic disorder, with delusions

292.12 Amphetamine induced psychotic disorder, with hallucinations

Cannabis induced psychotic disorder, with hallucinations

Cocaine induced psychotic disorder, with hallucinations

Hallucinogen induced psychotic disorder, with hallucinations

Inhalant induced psychotic disorder, with hallucinations

Opioid induced psychotic disorder, with hallucinations

Phencyclidine induced psychotic disorder, with hallucinations

Sedative, hypnotic, or anxiolytic induced psychotic disorder, with hallucinations

Other substance induced psychotic disorder, with hallucinations

291.89 Alcohol induced anxiety disorder

Alcohol induced bipolar and related disorder

Alcohol induced depressive disorder

Alcohol induced mild neurocognitive disorder

Alcohol induced sexual dysfunction

Alcohol induced sleep disorder

Alcohol induced mood disorder

291.10 Alcohol induced major neurocognitive disorder, amnestic confabulatory type

Alcohol induced persisting amnestic disorder

291.20 Alcohol induced major neurocognitive disorder, nonamnestic confabulatory type

Alcohol induced persisting dementia

291.00 Alcohol intoxication delirium

Alcohol withdrawal delirium

292.81 Medication-induced delirium

Opioid intoxication delirium

Other (or unknown) substance intoxication delirium

Hallucinogen intoxication delirium

Phencyclidine intoxication delirium

Sedative, hypnotic, or anxiolytic intoxication delirium

Sedative, hypnotic, or anxiolytic withdrawal delirium

Amphetamine (or other stimulant) intoxication delirium

Cannabis intoxication delirium

Cocaine intoxication delirium

Inhalant intoxication delirium

292.84 Amphetamine (or other stimulant) induced bipolar and related disorder

Amphetamine (or other stimulant) induced depressive disorder

Cocaine induced bipolar and related disorder

Cocaine induced depressive disorder

Inhalant induced depressive disorder

Opioid induced depressive disorder

Other (or unknown) substance induced bipolar and related disorder

Other (or unknown) substance induced depressive disorder

Other hallucinogen induced bipolar and related disorder

Other hallucinogen induced depressive disorder

Phencyclidine induced bipolar and related disorder

Phencyclidine induced depressive disorder

Sedative, hypnotic, or anxiolytic induced bipolar and related disorder

Sedative, hypnotic, or anxiolytic induced depressive disorder

Amphetamine induced mood disorder

Cocaine induced mood disorder

Hallucinogen induced mood disorder

Inhalant induced mood disorder

Opioid induced mood disorder

Phencyclidine induced mood disorder

Sedative, hypnotic, or anxiolytic induced mood disorder

Other substance induced mood disorder

292.89 Inhalant induced mild neurocognitive disorder

Opioid intoxication

Opioid induced anxiety disorder

Opioid induced sexual dysfunction

Other (or unknown) substance intoxication

Other (or unknown) substance induced anxiety disorder

Other (or unknown) substance induced mild neurocognitive disorder

Other (or unknown) substance induced obsessive-compulsive and related disorder

Other (or unknown) substance induced sexual dysfunction

Other hallucinogen intoxication

Other hallucinogen induced anxiety disorder

Phencyclidine intoxication

Phencyclidine-induced anxiety disorder

Sedative, hypnotic, or anxiolytic intoxication

Sedative, hypnotic, or anxiolytic nduced anxiety disorder

Sedative, hypnotic, or anxiolytic induced mild neurocognitive disorder

Sedative, hypnotic, or anxiolytic induced sexual dysfunction

Amphetamine (or other stimulant) induced anxiety disorder

Amphetamine (or other stimulant) induced obsessive-compulsive and related disorder

Amphetamine (or other stimulant) induced sexual dysfunction

Amphetamine or other stimulant intoxication

Caffeine induced anxiety disorder

Cannabis intoxication

Cannabis induced anxiety disorder

Cocaine intoxication

Cocaine induced anxiety disorder

Cocaine induced obsessive-compulsive and related disorder

Cocaine induced sexual dysfunction

Hallucinogen persisting perception disorder

Inhalant intoxication

Amphetamine (or other stimulant) induced sleep disorder

Caffeine induced sleep disorder

Cocaine induced sleep disorder

Inhalant induced anxiety disorder

Opioid induced sleep disorder

Sedative, hypnotic, or anxiolytic induced sleep disorder

Other (or unknown) substance induced sleep disorder

292.90 Amphetamine (or other stimulant) induced psychotic disorder

Cannabis induced psychotic disorder

Cocaine induced psychotic disorder

Inhalant induced psychotic disorder

Other (or unknown) substance induced psychotic disorder

Other hallucinogen induced psychotic disorder

Phencyclidine induced psychotic disorder

Sedative, hypnotic, or anxiolytic induced psychotic disorder

Unspecified caffeine related disorder

Unspecified cannabis related disorder

Unspecified hallucinogen related disorder

Unspecified inhalant related disorder

Unspecified opioid related disorder

Unspecified other (or unknown) substance related disorder

Unspecified phencyclidine related disorder

Unspecified sedative, hypnotic, or anxiolytic related disorder

Unspecified stimulant related disorder

Unspecified tobacco related disorder

Amphetamine related disorder NOS

Nicotine related disorder NOS

**Antisocial Personality Disorder**

301.70

**Borderline Personality Disorder**

301.83

**Narcissistic Personality Disorder**

301.81

**Cluster A Personality Disorders**

301.20 Schizoid personality disorder

301.22 Scizotypical personality disorder

301.00 Paranoid personality disorder

If cluster A is defined, but not the specific personality disorder, data is included in this category.

**Cluster B Personality Disorders**

301.50 Histornic personality disorder, is included in this category because of the very

low prevalence of the disorder within this sample.

If cluster B is defined, but not the specific personality disorder, data is included in this category.

**Other Personality Disorders**

301.90 Unspecified personality disorder

Personality disorder not otherwise specified

301.89 Otherwise specified personality disorder

The following categories were excluded from the network analysis bescause of a small sample size within the category (*n* <20).

**Gambling Disorder (*n =* 19)**

312.31 Gambling disorder

**Paraphelia (*n =* 16)**

302.20 Pedophilic disorder

Pedophilia

302.82 Voyeuristic disorder

293.83 Sexual masochism disorder

302.84 Sexual sadism disorder

302.89 Frotteuristic disorder

Other specified paraphilic disorder

302.90 Unspecified paraphilic disorder

Paraphilia NOS

**Cluster C Personality Disorders (*n =* 18)**

301.60 Dependent personality disorder

301.82 Avoidant personality disorder

301.40 Obsessive-compulsive personality disorder

If cluster C is defined, but not the specific personality disorder, data is included in this category.

**Mental Disorder due to Another Medical Condition (*n =* 14)**

293.00 Delirium due to another medical condition

Delirium due to multiple etiologies

293.81 Psychotic disorder due to another medical condition, with delusions

293.90 Mental disorder NOS due to general medical condition

294.00 Amnestic disorder due to general medical condition

294.80 Obsessive-compulsive and related disorder due to another medical condition

Other specified mental disorder due to another medical condition

294.90 Only if diagnosed under DSM-5: Unspecified mental disorder due to another

medical condition.

310.10 Personality change due to another medical condition

**Delirium (*n* = 2)**

780.09 Unspecified delirium

Other specified delirium

Delirium NOS

**Dissociative Disorders (*n* = 1)**

300.14 Dissociative identity disorder

300.15 Other specified dissociative disorder

Unspecified dissociative disorder

Dissociative disorder NOS

**Eating Disorders (*n* = 3)**

307.10 Anorexia nervosa

307.50 Unspecified feeding or eating disorder

Eating disorder NOS

**Gender Dysphoria (*n* = 6)**

302.60 Gender dysphoria in children

Other specified gender dysphoria

Unspecified gender dysphoria

Gender identity disorder in children

Gender identity disorder NOS

302.85 Gender dysphoria in adolescents and adults

**Sleep Disorder (*n* = 3)**

307.42 Primary insomnia

780.52 Insomnia disorder

Other specified insomnia disorder

Unspecified insomnia disorder

**Somatic Symptom Disorders (*n* = 7)**

300.11 Conversion disorder

300.82 Somatic symptom disorder

Unspecified somatic symptom and related disorder

Somatoform disorder NOS

307.89 Pain disorder associated with both psychological factors and a general medical

Condition

**Somatorform Disorders (*n* = 2)**

300.7 Body dysmorfic disorder

Illness anxiety disorder

Hypochondriasis

**Obsessive-Compulsive Disorder (*n* = 14)**

300.3 Obsessive-compulsive disorder

Other specified obsessive-compulsive and related disorder

Unspecified obsessive-compulsive and related disorder

Hoarding disorder

312.39 Trichotillomania

**Withdrawal (*n* = 15)**

291.81 Alcohol withdrawal

292.00 Amphetamine or other stimulant withdrawal

Caffeine withdrawal

Cannabis withdrawal

Cocaine withdrawal

Opioid withdrawal

Opioid withdrawal delirium

Other (or unknown) substance withdrawal

Other (or unknown) substance withdrawal delirium

Sedative, hypnotic, or anxiolytic withdrawal

Sedative, hypnotic, or anxiolytic withdrawal delirium

Tobacco withdrawal

**Movement Disorder (*n* = 4)**

293.89 Unspecified catatonia

Catatonia associated with another mental disorder

Catatonic disorder due to another medical condition

333.72 Medication induced acute dystonia

Tardive dystonia

333.85 Tardive dyskinesia

Finally, the category “Unspecified mental disorders” was excluded bacause of its indistinct nature, it provides no information on specific symptoms or behaviors related to the category. Keeping in mind the aim of the current study, to examine the relations between specific mental disorders and preoblematic substance use, the variable would not have contributed to this goal.

**Unspecified Mental Disorder (*n* = 24)**

300.90 Unspecified mental disorder

Other specified mental disorder

Mental disorder not otherwise specified

The merging of DSM-IV and DSM-V diagnoses resulted in 23 categories of mental disorders used in the network analysis of mental disorders.
