# Supplementary material for: Investigating the prevalence of problematic substance use and mental disorders in a large sample of prisoners with mental illness: network analysis
Source: BJPsych Open. 2023 Jul 6;9(4):e122. doi: 10.1192/bjo.2023.514 (PMC10375871; doi:10.1192/bjo.2023.514)
Supplement: Supplementary file 1 [file bjosup.zip › S2056472423005148sup002.docx]

**­­­Appendix B**

Three categories of problematic substance use are used in this study. Differentiation is based on the manual of the Historisch, Klinisch, Toekomst –Revisie (Historical, Clinical, Future – Revised; HKT‐ R),(1) and identification of hard drugs under the Dutch law.(2,3)

**Common substances of abuse encountered in clinical practice, classified as *hard drugs***

- Opiates and derivatives (heroin, fentanyl, morphine and oxycodone)
- Psychostimulants (cocaine, amphetamine and derivatives, cathinone and derivatives)
- Hallucinogens and dissociatives (ketamine, psilocybin and lysergic acid diethylamine)
- Sedatives (benzodiazepines and gamma hydroxy butyrate)

**References**

1. Spreen M, Brand E, Horst P Ter, Bogaerts S. Handleiding en Methodologische Verantwoording HKT-R, Historisch, Klinische en Toekomstige – Revisie [Guidlines and Methodological Research of the HKT-R, Historical, Clinical and Future – Revision]. Groningen, The Netherlands: Dr. van Mesdag kliniek; 2014

2. wetten.nl - Regeling - Opiumwet - BWBR0001941 [Internet]. Available from: https://wetten.overheid.nl/BWBR0001941/2020-01-01#BijlageI. [Cited 2021 Feb 28].

3. wetten.nl - Regeling - Opiumwet - BWBR0001941 [Internet]. Available from: https://wetten.overheid.nl/BWBR0001941/2020-01-01#BijlageII. [Cited 2021 Feb 28].
